# Between-group competition, intra-group cooperation and relative performance

**DOI:** 10.3389/fnbeh.2015.00033

**Published:** 2015-02-17

**Authors:** Juan C. Cárdenas, César Mantilla

**Affiliations:** ^1^Economics Department, Universidad de los AndesBogotá, Colombia; ^2^Institute for Advanced Study in ToulouseToulouse, France

**Keywords:** laboratory experiment, public goods games, social comparison, intra-group competition, individual and group performance

## Abstract

We report the results of a new public goods experiment with an intra-group cooperation dilemma and inter-group competition. In our design subjects receive information about their relative individual and group performance after each round with non-incentivized and then incentivized group competition. We found that, on average, individuals with low relative performance reduce their contributions to the public good, but groups with low performance increase theirs. With incentivized competition, where the relative ranking of the group increases individual payoffs, the reaction to relative performance is larger with individuals contributing more to the group; further, we observe that the variance of strategies decreases as individual and group rankings increase. These results offer new insights on how social comparison shapes similar reactions in games with different incentives for group performance and how competition and cooperation can influence each other.

## 1. Introduction

Collective action most likely evolved as a survival group strategy to overcome challenges and threats difficult to surpass individually. Achieving collective action, however, requires solving the problem of incentives within the group, namely, the conflict among individuals who would be better materially if they reap the benefits of cooperation by others but do not assume the cost. Groups with higher levels of cooperation, on the other hand, could reproduce their strategies more successfully making them more competitive against other groups. This competition among groups over scarce resources decreases the within-group conflict at the cost of raising the between-group conflict^1^.

One particular condition shaping competition is the availability of information on individual and group performance. When these informational sets are independently provided, the feedback at the group level decreases the salience of selfish incentives, increasing within-group cooperation (Burton-Chellew and West, 2012) at the cost of additional between-group conflict. However, subjects' reaction to the simultaneous provision of individual and group ranking has been rather unexplored. By receiving simultaneous feedback on individual and group performance subjects may develop richer responses to their relative success with respect to other group members but also to their group's success with respect to other groups, especially in presence of competition for additional resources. These different incentives bring a complex interaction of cooperation and conflict. One individual's higher relative performance could increase her individual payoffs at the expense of reducing the relative performance of her group, and thus harming the group's relative performance which in turn would decrease her individual payoffs.

The earliest experimental evidence from the effects of intergroup competition, including the development of in-group and out-group dimensions and the increase of cooperativeness (resp. hostility) with subjects from the same (resp. the other) group, comes from the “Robber's Cave” experiment described in Sherif et al. (1954/1961). Incentivized experiments with a game theoretical foundation appear years after, showing that between-group competition reduces the within-group conflict and positively affect the cooperation levels (Rapoport and Bornstein, 1987; Bornstein, 1992; Erev et al., 1993; Bornstein and Ben-Yossef, 1994). In this second wave of experiments on group competition the term “discontinuity effect” was coined, meaning that the interactions between two unitary groups are more competitive than the interactions between two individuals (Schopler and Insko, 1992). These advances in the empirical literature were accompanied by theoretical models describing the conditions in which cooperation may emerge under a scenario of between-group competition for a share of the resource jointly produced (Hausken, 1995, 2000)^2^.

Bornstein's empirical work was followed by new experiments involving between-group competition in contest games and public goods games^3^, both characterized by a tension between collective and individual incentives. In contest games, fixed groups compete for a reward to be allocated based on the relative investments in the contest (Abbink et al., 2012; Leibbrandt and Saaksvuori, 2012). As the investments are dissipated after the prize allocation, the socially desirable contributions to the contest are minimal.

When between-group competition is embedded in a public goods game the within-group conflict is reduced at the cost of an increased salience of between-group conflict. Contributions provide a benefit to all group members, including the contributor, which is smaller than the benefit of not contributing. As the between-group conflict is not inherent to the conventional public goods game, such dimension is introduced through rewards based on group performance. For the different rules used in the studies below, individual contributions are positively affected by competition. In Gunnthorsdottir and Rapoport (2006) and Burton-Chellew et al. (2010) groups compete for additional monetary units (MUs). In the former, a unique prize is shared evenly or proportionally across treatments. In the latter, multiple prizes are offered, ranging from 80% of the endowment for the top performer, to 10% of the endowment for the bottom performer. In Tan and Bolle (2007) two groups compete based on their aggregate contributions for a higher return rate of the common fund. In Puurtinen and Mappes (2009) two out of six groups are randomly drawn each round to compete. All competitors receive twice the difference of the aggregate contribution of each group, a positive transfer for the winners deducted from the losers' payoffs. In Reuben and Tyran (2010) groups compete for being ranked first, a position that may be simultaneously reached by all groups. Groups below the first place face a reduction of their payoffs proportional to their ranking position. Burton-Chellew and West (2012) claim that, as a consequence of the long-term group selection process, people react to cues of competition in the absence of material benefits. In a public goods experiment they provide information on the between-group performance to the treatment group and information on the within-group performance to a control group. Their findings indicate that contributions are larger after receiving between-group's feedback. In Markussen et al. (2014) the group ranked first receives a transfer from the group ranked last (with the group in-between having unaltered payoffs). Subjects collectively decide whether to introduce competition or not using two different electoral rules: absolute majority or group veto. Once again, competition increases contribution levels. Although this rule is very likely to be elected under the majority rule, it is implemented less than half of the times under group veto. In most of the public goods games with group competition subjects are randomly rematched after each interaction. The exceptions are Reuben and Tyran (2010) and Markussen et al. (2014) who hold the groups' composition constant; and (Tan and Bolle, 2007), who introduce group competition using both matching protocols.

The increase in the salience of between-group competition does not necessarily modify the equilibrium of the game, under the assumption of players that maximize only their personal material payoffs. The most evident case is Burton-Chellew et al. (2010), where only the informational sets vary between treatments. But in Tan and Bolle (2007) and Puurtinen and Mappes (2009) null contribution is also the only equilibrium despite the incentives to compete. In Gunnthorsdottir and Rapoport (2006); Burton-Chellew et al. (2010) and Markussen et al. (2014) the a positive contribution level (lower than the endowment) becomes the unique equilibrium of the game. Finally, in Reuben and Tyran (2010) null contribution and full contribution are equilibria of the game.

We propose an experimental design where *N* subjects, divided in fixed groups of size *N*/6, play a repeated public goods game within the group while they compete against other groups for a ranking position mapping in a payoffs' multiplier. The unique symmetric equilibrium in our game is full contribution. We want to explore how the relative standing or ranking of individuals within groups and the ranking of one's group relative to other groups matter for changing behavior. In our setting players know their relative performance both individually and as a group. In the first stage individuals learn about their relative performance, individually and group wise, but payoffs are not affected. In the second stage, besides informing them of their relative performance, the players' payoffs are multiplied by a factor μ that increases linearly with the position of their group in the ranking. Subjects in the bottom three groups see their payoffs reduced, whereas those subjects in the top three groups see their payoffs increased. The intensity of between-group competition shifts the game theoretical Nash prediction from zero contributions to the public good to full cooperation. The efficiency of the symmetric equilibrium makes this institution, at least in theory, *ex ante* attractive if it is assumed that all subjects are equally likely to perform under incentivized competition. In practice, it allows exploring heterogeneous beliefs and responses to competition because downward deviations are rational only if subjects believe that all the groups are not equally competitive. We also explore the role of group size under the hypothesis that the likelihood to coordinate and the salience of between-group conflict are higher for smaller groups.

In this paper we contribute to the group competition literature in two different fronts. First, by exploring in detail the informational effects of individual and group performance on between-group competition. We find that while the relative group standing in the previous round decreased current cooperation (i.e., lower contributions), the relative individual standing had the opposite effect. Second, by showing that these informational effects are structurally similar with or without additional material incentives to compete. The only difference is that the intercept is shifted upwards (i.e., toward higher contribution levels) when the payoffs are affected by the group's ranking.

These results show a complex dynamic regarding the problem of between-group competition with intra-group cooperation, and opens further questions regarding rewards at the individual and group levels.

## 2. Methods

The experiment was conducted between September and November 2012 at Universidad de los Andes (Bogotá, Colombia) using z-Tree (Fischbacher, 2007). Informed consent was obtained from all the participants, who were recruited through email solicitations. Due to the low-risk nature of this experiment, the voluntary participation of subjects, and the use of a consent form signed by them, approval from the ethics committee was not requested. However, the protocol followed all ethical standars common in economic experiments. Each session lasted between 70 and 90 min. Participants earned on average $27,900 COP (Colombian pesos), about $15.3 usd, including a show-up fee of $2000. This payment is about 1.5 times the Colombian minimum daily wage at the time of the experiment^4^.

In each session, participants were divided into six groups to play a voluntary contributions mechanism (VCM) for twenty rounds. We assigned players to groups of either 3, 4, or 5 people and remained fixed during the whole session. Incentives including group performance were introduced in the last ten rounds, although information on individual and social comparison were provided after each round for the entire session. Participants were endowed with 10 tokens (*e* = 10) in each round. They had to decide how many tokens they wanted to allocate in a common fund (*x_i_* ϵ {0, 1, 2, …, 10}), keeping the rest in a private fund. Each token in the common fund had a payoff of $150 COP to be divided equally among the group members, independently of the individual contributions. Each token kept in the private fund generated $100 COP to the individual player. This information was known by all subjects.

At the end of each round participants were reminded of their contribution, and were informed about their group's aggregate contribution, their round earnings, their individual ranking among the whole population in the session (but not within their group)^5^, their group ranking (based on aggregate earnings), and the highest and lowest earnings of the round among all subjects in the session.

The 20 rounds were divided into two stages of ten rounds each. We will call the first stage *pseudocompetition* because payoffs are not directly affected by the group's ranking. In this stage, comprising rounds 1–10, we have a standard VCM payoffs function

$$\pi_{i}^{0}=(e-x_{i})+\alpha \sum_{i=1}^{N}x_{i}$$

with a unique Nash equilibrium (NE) in *x_i_* = 0.

In the *competition* stage, comprising rounds 11–20, earnings are multiplied by a factor μ function of the group's rank *r_k_* as shown in Table 1. A multiplier μ = 2.0 is given to the best ranked group, and it decreases linearly by a factor of 0.4 with each position in the group ranking. In the case of a tie between two or more groups, their multipliers are averaged. With the multiplier μ, the payoffs function becomes

$$\pi_{i}^{1}=\mu (r_{k})\left((e-x_{i})+\alpha \sum_{i=1}^{N}x_{i}\right).$$

**Table 1:** **Payoff multipliers according to group performance**.

**Group Ranking (*r_k_*)**	**Bottom**			**Top**		
	**1**	**2**	**3**	**4**	**5**	**6**
Multiplier (μ)	0.0	0.4	0.8	1.2	1.6	2.0

The entire payoffs function is multiplied by μ to hold constant the ratio between the returns of the private and the common fund. It prevents that contributing becomes increasingly attractive with higher expectations of group performance. A similar incentive for group performance is proposed in Reuben and Tyran (2010), with multipliers that are used for punishing groups not able to rank first. Under their conditions the game has two symmetric equilibria: *null contribution* and *full contribution*. In our experimental design the only symmetric NE is *full contribution* because the multiplier rewards the “top performers” and punishes the “bottom performers” simultaneously. To see why, consider that all subjects are contributing *x* and thus all groups are tied (μ = 1.0). A subject will have incentives to contribute an additional unit and make her group rank first (μ = 2.0) if

2(e−(x+1)+α(nx+1))>(e−x)+αnx

which is strictly positive for every *x* ϵ {0, …, 9}. Once they reach *x* = 10, any deviation will send the group to the bottom of the ranking with a null multiplier, ratifying the lack of incentives to deviate from *x* = 10. As in Reuben and Tyran (2010), in our experimental setting the between-group incentives lead to some asymmetric equilibria with different contribution levels across groups. They appear when a single subject cannot alter the group's ranking given (her beliefs about) the other subjects' actions. Consider a very simple case in which player *i* expects that at least one of her group mates will not contribute her full endowment, but she simultaneously expects that in all the other groups subjects will behave as predicted in the symmetric equilibrium (*x* = 10). This is equivalent to expect a multiplier μ = 0, for which all the pure strategies turned out to be an equilibrium of the game. Subjects from other groups, who are contributing *x* = 10, do not have incentives to deviate unilaterally. Their current payoff (1.2 α*ne*) is larger than what they could get by keeping all their endowment and rank fifth as group (0.4(*e* + α (*n* − 1)*e*)), the best scenario they could consider to defect.

Group size is an additional source of variation in our experimental design. We hypothesize that smaller groups are more reactive to the cues of between-group competition (and therefore that they will be closer to the NE under competition) for two reasons: first, the intra-group coordination required to raise the group's relative performance might be, in theory, easier to achieve (e.g., through simpler computations of intra-group and inter-group beliefs); second, in smaller groups the salience of the within-group conflict might decrease more drastically with competition.

Each unit in the common fund generated the same value irrespective of the group size. Therefore, the marginal per capita return of the fund (MPCR) α depends then on the number of participants in the session. We conducted two sessions with 3 players per group (α = 0.500), two sessions with 4 players per group (α = 0.375), and another two sessions with 5 players per group (α = 0.300) for a total of 6 sessions and 144 subjects.

Instructions were given orally and in written form. The instructions were followed by two examples on the board. The protocol read to the participants is included in the online Supplementary Material. After the tenth round new instructions were given for the *competition* stage. The new instructions emphasized the ranking calculations and transformation of the payoffs. A post experimental survey was conducted to collect data on participants' competitiveness and trustworthiness, as well as basic demographics. The questions used to measure competitiveness are shown in the online Supplementary Material.

## 3. Results

### 3.1. Cooperation with and without material incentives for competition

**Figure 1:**
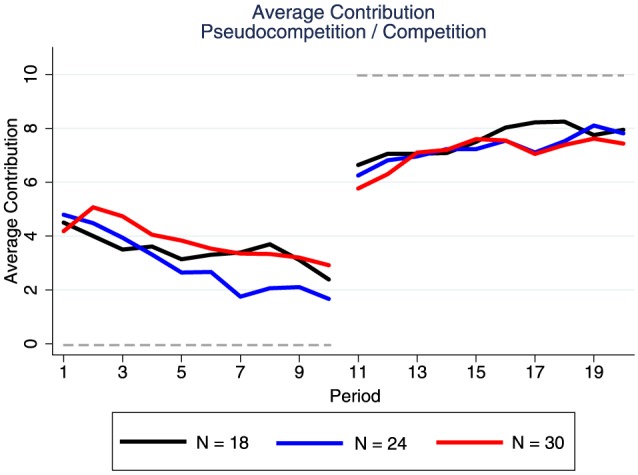
**Average contribution with and without group competition**. The gray horizontal line represent the Nash equilibrium of the game in the *pseudocompetition* and the *competition* stages.

In the *pseudocompetition* stage the average contribution was 3.4 tokens. We observe (see Figure 1) the decay typical of a standard linear PG game (Ledyard, 1995; Zelmer, 2003), ranging from 4.5 tokens in round 1–2.4 tokens in round 10. As expected from the theoretical predictions, contribution levels are higher once the between-group competition is materially incentivized. In the *competition* stage the average contribution is 7.3 tokens. The increasing contribution rate, going from 6.1 tokens in round 11–7.7 tokens in round 20, suggests that subjects learn about the incentives of the game with experience but they are still far from the symmetric NE prediction.

We do not find an effect of group size, possibly because we primed the total value of each token in the common fund (invariant with group size) instead of priming the value of the MPCR (which decreases with group size). We also find that the percentage of subjects following the NE *x* = 0 goes from 11% in round 1 to 36% in round 10. When the NE is *x* = 10 the increase of players following this strategy goes from 31% in round 11 to 55% in round 20. The NE outcome under competition, i.e., maximum contribution levels for all participants, did not emerge in any round of any session. However, smaller groups were more likely to maximize their total contribution to the common fund^6^.

**Figure 2:**
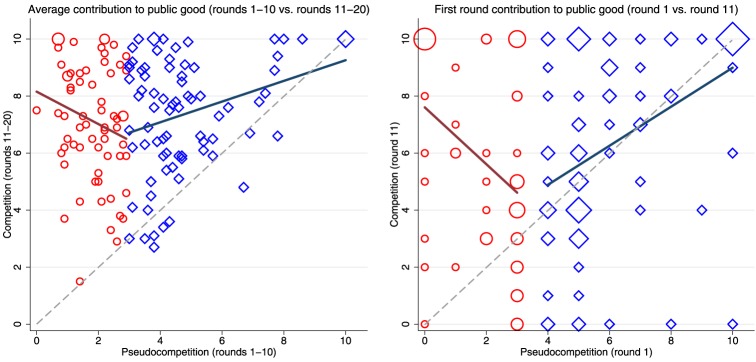
**Total contribution (per player) with and without competition**. Participants are classified according to the median average contribution (*x* = 3) under *pseudocompetition* (rounds 1–10). The red circles represent participants below the median (“low contributors”) and blue diamonds represent participants above the median (“high contributors”). In the left panel we plot the mean contribution per player under *pseudocompetition* vs. the mean contribution under *competition*. For the “low contributors” the correlation between the average contribution across the two stages is negative and marginally significant (–0.21, *p*-value 0.092), whereas for the “high contributors” this correlation is positive and significant (0.31, *p*-value 0.006). In the right panel we plot the contribution of each player in the first round of each treatment showing again the opposite relationships for low and high contributors (median contribution in round 1 is *x* = 4). Marker size reflects the frequency of observations in each point of the grid.

The fact that the Nash equilibrium is played more often with material incentives for competition (39%) than without them (21%) is not surprising since the most self-regarding and the more cooperative subjects in the first stage react similarly to incentivized competition, as is evidenced in Figure 2. A shift in individualists' cooperative behavior, triggered by intergroup competition, is reported in Probst et al. (1999). Their test-based measure of individualism (compared to collectivism) is correlated with more contributions in a public goods game with intergroup dependent payoffs^7^.

On the left panel of Figure 2 is observed that a small proportion of subjects (5.6%) contribute more without than with incentivized competition. We will call then the “non-competitors” because we argue that this is due to their different preferences for performing in a competitive environment (Niederle and Vesterlund, 2007; Niederle et al., 2013). For this small fraction of non-competitors the proportion of women is much higher (50% compared to 36% among the competitors, *p*-value 0.429), they are much more likely to report that they prefer to play without than with competition (75% compared to 33% among the competitors, *p*-value 0.016), and they display less competitive attitudes according to a standardized principal components index constructed using six questions responded in the post-experimental survey (0.48 standard deviations less competitive, *p*-value 0.160). However, the statistical tests are not very likely to result significant given the low proportion of non-competitors.

### 3.2. Responses to feedback on individual and group performance

**Figure 3:**
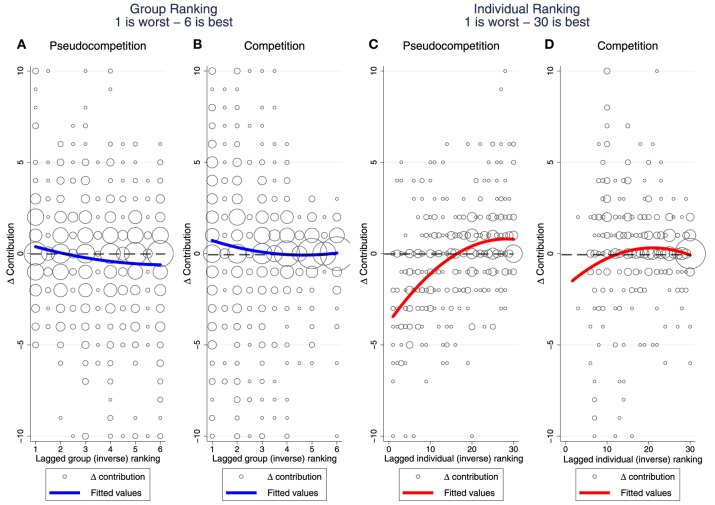
**Changes in contribution with respect to relative performance**. **(A,B)** show the negative effect of group ranking in Δ*Contribution* for the *pseudocompetition* and *competition* stages, respectively. **(C,D)** show the positive effect of individual ranking in Δ*Contribution* for the *pseudocompetition* and *competition* stages, respectively. For treatments with 18 and 24 participants the individual ranking was rescaled to match the treatment with 30 participants. In **(C,D)** subjects in the worst performing group are excluded given their null payoff, leading to a large number of ties at the bottom of individual ranking. Fitted values refer to a quadratic regression in which the independent variables are the respective ranking (group or individual) and its square, and the dependent variable is Δ*Contribution*.

We show in Figure 3 the effect of feedback on group and individual performance in subsequent contribution decisions. A comparison of the four panels in this figure reveals the following three patterns: (i) Better group performance reduces individual contributions, whereas better individual performance increases individual contributions. (ii) The response to group performance shifts upwards with incentivized competition and, as a consequence, subjects in top-performing groups stop reducing their contribution. On the other hand, the nonlinearity in the response to individual performance becomes less pronounced with incentivized competition. (iii) The variance of responses to feedback on individual and group performance decreases with ranking positions.

We use a fixed effects estimator (demeaned variables method) to test patterns (i) and (ii) by regressing Δ*Contribution* on individual and group ranking's in the quadratic polynomial form^8^. This method allows us to control for unobserved time invariant characteristics of the subjects. We observe the negative (and decreasing) effect of group ranking as well as the positive (and decreasing) effect of individual ranking before and after introducing material incentives to compete. We also run the regressions excluding the participants belonging to the group that end up last in each round. Our motivation for this robustness check is twofold: to check that the nonlinearities are not driven by subjects in groups that ranked last, who might be different from the other participants; and to exclude from the subsample the subjects with perfect correlation between individual and group ranking given their null payoff under *competition*. The nonlinear effect of group ranking is no longer significant under *pseudocompetition*, whereas the coefficients increase in magnitude under *competition*. This result suggests that the incentivized inter-group competition induces more responsiveness to the social comparison, if compared to the *pseudocompetition*.

**Figure 4:**
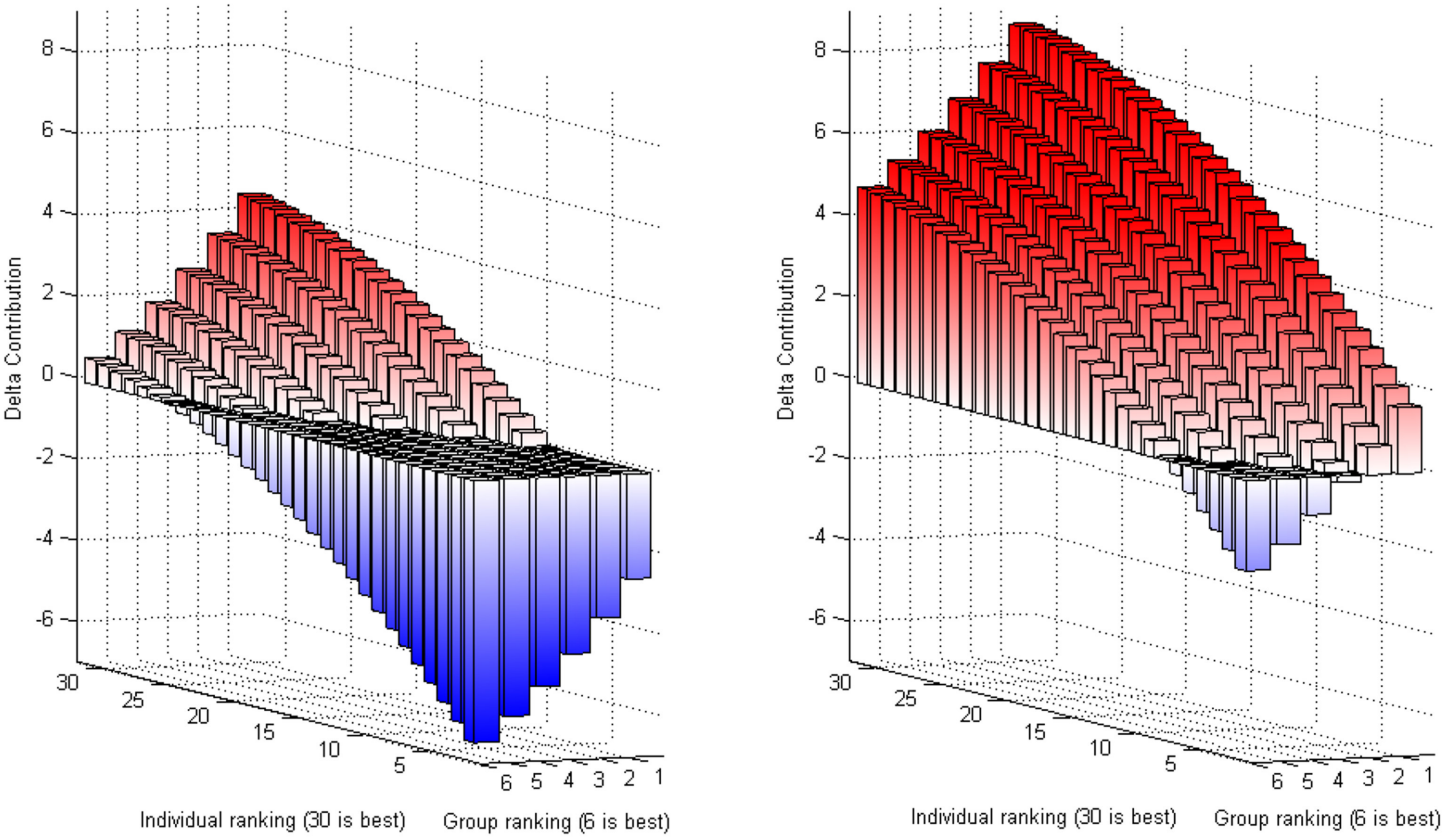
**Predicted change in contribution as a function of group and individual performance**. The left and right panels correspond to the predicted difference in contribution under *pseudocompetition* and *competition* using results from columns (1) and (3) in Table 2, respectively. Red (resp. blue) bars correspond to a positive (negative) value of Δ*Contribution*.

The predicted response Δ*Contribution* for each combination of group and individual ranking is plotted in Figure 4. The same pattern is observed across treatments: high individual performance and low group performance are followed by an increase in contributions. The stark difference is a positive shift in the intercept once group competition is materially incentivized. Under *competition* the individual contribution is slightly reduced only in case of a high group ranking (at least 5) and a low individual ranking (at most 4). This reaction could be interpreted as a reciprocal response to the environmental cues suggesting higher contributions with respect to the other team members.

We perform three robustness checks to the regressions shown in Table 2. The results are reported in the online Supplementary Material. First, we show that all the coefficients are statistically different between treatments (see Table B.1 in Supplementary Material). Second, we compute again the regressions excluding the observations in which subjects were playing the NE, i.e., those constrained to alter their extraction level. As shown in Supplementary Material (Table B.2) our estimates are robust in the *competition* stage, although the quadratic effect of group ranking disappears under *pseudocompetition*. Third, we run the regression separately for each group size. The coefficients are reported in Supplementary Material (Table B.3). The marginally decreasing (increasing) effect of group (individual) ranking is captured in the regression for each subsample, but the statistical significance of the coefficients is compromised due to our loss of statistical power. This problem is particularly stark in the *pseudocompetition* stage for all group sizes, and in the *competition* stage for the smaller group size.

**Table 2:** **Fixed effects regression: ΔContribution explained by individual and group rankings**.

**Dependent variable:**	**Pseudocompetition**		**Competition**	
**Δ Contribution**	**(1)**	**(2)**	**(3)**	**(4)**
Lagged group ranking	−1.045^***^	−0.661^**^	−6.912^***^	−9.666^***^
	(0.106)	(0.244)	(1.012)	(0.934)
Lagged group ranking squared	0.039^***^	−0.008	0.481^***^	0.765^***^
	(0.009)	(0.025)	(0.112)	(0.108)
Lagged individual ranking	0.429^***^	0.408^***^	1.405^***^	1.738^***^
	(0.053)	(0.059)	(0.188)	(0.173)
Lagged individual ranking squared	−0.006^**^	−0.005^**^	−0.023^***^	−0.029^***^
	(0.002)	(0.002)	(0.004)	(0.004)
Round	−0.013	−0.019	−0.089^***^	−0.083^**^
	(0.024)	(0.022)	(0.020)	(0.028)
Constant	−1.998^***^	−2.453^**^	2.215^***^	4.643^***^
	(0.490)	(0.810)	(0.448)	(0.388)
Group ranked at bottom excluded	No	Yes	No	Yes
Observations	1,296	1,101	1,296	1,101
R-squared	0.400	0.402	0.153	0.228
Number of subjects	144	144	144	144

*Standard errors were computed clustering observations at the session level. For treatments with 18 and 24 participants the individual ranking was rescaled to match the treatment with 30 participants. The *Round* variable goes from 2 to 10 in all regressions. Standard errors reported in parentheses*.

*** *p < 0.01*,

** *p < 0.05*.

The differences in within-ranking variance between stages described in pattern (iii) were tested statistically (see Table B.4 in the online Supplementary Material). The variance in Δ*Contribution* decreases under *competition* when the group or the individual ranking increases, whereas the variance remains relatively constant under *pseudocompetition*.

## 4. Discussion

We propose a VCM game in which the introduction of incentivized between-group competition alters the Nash equilibrium of the game from null contribution to full contribution. This manipulation drastically increases within-group cooperation, as reported by others where competition induced higher contributions in equilibrium (Gunnthorsdottir and Rapoport, 2006; Reuben and Tyran, 2010). Regarding the responses to feedback, the negative effect of group ranking (particularly under *pseudocompetition*) is similar to the findings in Tan and Bolle (2007), in which members from the winning (resp. losing) group tend to decrease (resp. increase) their contribution level.

The distance to the equilibrium prediction in the *pseudocompetition* stage has been explained by conditional cooperation (Chaudhuri, 2011), the intention to establish efficiency enhancing norms (Andreoni, 1988) and bounded rationality (Ledyard, 1995; Kummerli et al., 2010). However, only the latter explanation can be successfully extrapolated to the analysis of the *competition* stage. We consider four non mutually exclusive explanations for this behavior. First, subjects might be following the asymmetric equilibria aforementioned. Remember that if subjects consider that not all groups are equally competitive they may have incentives to set their contribution levels below the NE. This is very plausible since players are getting information in each round about their individual and group performance. Further, (Markussen et al., 2014) provide evidence of subjects correctly expecting that their group was likely to be outcompeted (and therefore they voted against introducing competition). Assuming that subjects in our experiment were able to form similar expectations based on their past interactions, one should expect more diverse responses among those subjects in groups ranked last in the previous round. The decreasing variance of Δ*Contribution* with group ranking provides evidence in favor of this behavior.

A second explanation is that subjects might be confused due to the complexity of a simultaneous computation of in-group and out-group beliefs when subjects know that the groups are not equally good in the competition. If this is the case, subjects may develop rules of thumb based on the logic of the asymmetric equilibrium described above: subjects might underestimate the probability that their group will be reached by a group positioned below, and therefore they may opt for not contributing all their endowment. A decrease in the degree of confusion (possibly through a learning process) fits the increasing tendency of contributions over time observed in the competition stage.

A third possibility is that subjects are indirectly punishing low contributors in their group through a reduction in their contribution level^9^. We test econometrically the existence of reciprocal behavior by regressing Δ*Contribution* on the lagged average contribution from the other group members (results not shown). We limit our sample to those cases in which player *i* was contributing more than the average of her fellow group members (i.e., those in position to punish low contributors). We find that higher contributions from other group members increase contributions under *competition*. This is true for the full sample and for the subsample of top performing groups (ranked 4–6), but not for the subsample of bottom performing groups (ranked 1–3). Nonetheless, as the coefficient is only significant for the full sample, the punishment-based explanation is not entirely convincing.

One may think of an additional explanation based on the previous history of decay under *pseudocompetition*. Although we cannot test this hypothesis because all subjects faced both treatments in the same order, we argue that the history of the game is not very likely to explain the underinvestment in the common fund for two reasons. First, previous experiments did not find any order effect (Burton-Chellew et al., 2010; Burton-Chellew and West, 2012), or the effect implies that subjects contribute more under between-group competition when it is preceded by a control stage without competition (Puurtinen and Mappes, 2009). Second, as is shown in Figure 2 the relationship between the contribution with and without competition for a given player is U-shaped rather than monotonic. Therefore, it is hard to think about an unequivocal effect of previous cooperative decay.

We conclude highlighting the important role of the feedback of individual and group performance on future contributions. In particular, we want to stress that subjects are decreasing (resp. increasing) their contribution levels in response to high group (resp. individual) ranking, independently of the presence of material incentives to compete. The additional rewards (and penalties) incentivizing competition raise the average contribution levels, but they do not seem to alter the structural reaction to the environmental cues from relative performance. If we consider the complexity of updating beliefs at the in-group and out-group levels simultaneously, the similarities across treatments (despite the different NE) found in this work suggest the use of rules of thumb to process the type of information that triggers competition through social comparison. Given our conjecture about the importance of preferences for performing in a competitive environment in explaining the deviations from the equilibrium strategy, and the evidence on voting against competition found in Markussen et al. (2014), future research could involve cross-cultural differences in these preferences to test the reactiveness to the cues of competition, or manipulations in framings to evaluate the responsiveness to the incentives to compete and the incentives to cooperate.

### Conflict of interest statement

The authors declare that the research was conducted in the absence of any commercial or financial relationships that could be construed as a potential conflict of interest.

## Footnotes

^1^The study of multi-level selection has been witnessing a revival in natural and social sciences. See for instance (Sloan Wilson and Wilson, 2007; Bowles and Gintis, 2011).

^2^For instance in Hausken (2000), cooperative behavior might be sustained if the two groups are sufficiently closer in their initial cooperation levels, otherwise the less cooperative group does not have incentives to engage in competition.

^3^The standard voluntary contribution mechanism has been extensively used in the exploration of other-regarding preferences. Two surveys of the relevant literature in public good games are Ledyard (1995) and Chaudhuri (2011). For a meta-analysis see Zelmer (2003).

^4^1 USD = 1825 COP on November, 2012. Colombian daily minimum wage was $18,890 COP in 2012.

^5^In Burton-Chellew and West (2012) the information of individual ranking is given at the within-group level, along with the disaggregated individual contributions.

^6^With group size *n* = 5 only one group played the NE strategy for a single round in the *competition* treatment. When the group size was *n* = 4 only one group played the NE for four consecutive rounds (15–18). The NE strategy was played more often with groups formed by three players: In one of the sessions, in the last six rounds at least one group fully contributed (although it was not always the same group). In round 20 four groups played this strategy. In the other session, two groups played the NE eight times between rounds 12 and 20. A third group also played this strategy in round 14. For groups of three players the deviation from the NE is less likely to occur than for bigger groups: Only one of the five groups that reached the NE at least twice deviated from full contribution more than once (and there was only one group that deviates in a given round but rapidly corrected this mistake).

^7^We consider as individualistic subjects those with low contribution levels (i.e., below the median) in the *pseudocompetition* stage. See the legend in Figure 2 for more details.

^8^In theory, the variable Δ*Contribution* is subject to a truncation problem as it is bounded between −10 and 10. In practice, less than 2% of the observations are located in any of these extreme values of Δ*Contribution*. This is true for the *pseudocompetition* and the *competition* treatments.

^9^This a standard explanation for the decay of contributions in standard public goods games that is related to other-regarding preferences.

## Supplementary material

### Data availability

Experimental data, the programming of the statistical procedures and the experimental instructions are available in the following link: http://goo.gl/Sd0gWd
