# Supplementary material for: Between-group competition, intra-group cooperation and relative performance
Source: Front Behav Neurosci. 2015 Feb 17;9:33. doi: 10.3389/fnbeh.2015.00033 (PMC4330795; doi:10.3389/fnbeh.2015.00033)
Supplement: Supplementary file 1 [file Table1.PDF]

Frontiers in Behavioral Neuroscience

Research Article
21 January 2015

Supplementary Material: Between-group competition, intra-group cooperation and relative performance

Juan Camilo Cárdenas [1,*], **César Mantilla** [2]

[1] *Economics Department, Universidad de los Andes, Bogotá, Colombia*
[2] *Institute for Advanced Study in Toulouse (IAST), Toulouse, France*

Correspondence*:
Juan Camilo Cárdenas
Economics Department, Universidad de los Andes, Calle 19A No. 1-37 Este
Bloque W, Bogotá, Colombia, jccarden@uniandes.edu.co

1 EXPERIMENTAL INSTRUCTIONS

Instructions were displayed and read in Spanish to participants. Numbers in bold vary according to the treatment (group size).

Written Instructions: Stage 1

Welcome.

Thank you for your attendance. By coming to this activity, you have already earned $2,000 that will delivered to you with your final earnings. The activity in which you are about to participate aims to explore the economic decisions taken by individuals.

You belong to a group of **4** participants out of a total of **24** participants that are in this room at the moment. The identity of your group members will not be revealed at any time, during or after the exercise. The individual decisions of the other participants will not be revealed neither.

In this exercise you will receive 10 tokens in each period. You will decide to invest them in a private or in a common fund. For each token invested in the private fund, you will earn $100. This amount will be received only by you. Each token invested in the common fund by you or another group member, will generate $150 in earnings to be splitted equally among your **4** team members. Independently of your investment level in the common fund, you will receive a **fourth** of the total earnings in this fund. The more tokens in the common fund, the higher the earnings to be distributed among the group. The more tokens in the private fund, the higher your earnings.

Let's see an example before starting:

Suppose you contributed 4 tokens to the common fund and kept 6 tokens in the private fund. Also suppose that the other team members contributed a total of 10 tokens to the common fund.

You earnings correspond to $100 for each token in the private fund, a sum of $600, plus one**fourth** of the $2,100 produced by the 14 tokens in the common fund, a sum of $525. Therefore, your total earnings in this round are $1,150.

This exercise has a total of 20 periods divided into 2 stages, with 10 periods each. At the beginning of the second stage, groups will be reshuffled and a new rule will be announced and incorporated into the game.

If at this moment you have questions, please do not hesitate to raise your hand to clarify your doubt. If instructions are clear, and you confirm you are interested in taking part in this activity, please read and sign the informed consent form that will be delivered by the monitor.

Oral Instructions: Stage 1

Let's verify that instructions were properly understood. Remember you belong to a group of 4 subjects of a total of 24 participants. The group will remain fixed for the first 10 periods. For the last 10 periods you will be reallocated into another group of the same size. In each period you will receive 10 tokens, that could be invested in a private fund -that generates benefits only for you- or in a common fund -that generates earnings for you and the other three group members-.

Each token invested into the private fund will give you $100, while each token invested by you or another group member into the common fund will generate $150 to be splitted equally among the group members. Let us show another couple of examples [The monitor shows in the board another two predefined examples].

Instructions: Stage 2

For the second stage of this exercise a new rule will be introduced. Groups will be reshuffled, which means that the participants in your group will not be the same than in the first stage. This new group will remain the same for the next 10 periods.

From now on, your round earnings will be affected by a multiplier defined by your GROUP RANKING. The GROUP RANKING is calculated according to your group's total earnings in the round. The higher your group's earnings, the higher the multiplier.

The table on the right panel on your screen shows the multiplier effect for each ranking position (see Table 1 in the paper). Please take into account that the GROUP RANKING will be recalculated after each period.

To calculate the ranking your earnings are added to the earnings of the other group members. For example, suppose your earnings are $1,300, your group total earnings are $3,500, enough to be the group with the second highest earnings. Therefore, your earnings are multiplied by 1.6, and you will obtain $2,080 in that given period.

Oral Instructions: Stage 2

Let's verify that the new rule introduced for the second stage has been understood. Now the group ranking, defined by the sum of the earnings of all the group members, will define the round payment according to the table observed in the screen.

It's important to clarify that in case of a tie between two or more groups, the multipliers of all the tied groups will be averaged. Suppose for example that groups in second and third place are tied. The position shown in the screen will be 2.5 and the earnings multiplier will be 1.4 for both groups, the average value of the second and third multipliers. Let us show another example [The monitor shows in the board the case of a tie between all six groups].

Figure 1. Screenshot of the feedback received after every round from the *competition* stage (in Spanish). The only difference with respect to the feedback received after every round from the *pseudocompetition* stage is that earnings are displayed only once (instead of the earnings before and after the multiplier). The English translation of each one of these lines, in the same order as they are displayed, is as follows: *(1) The number of TOKENS YOU INVESTED IN THE COMMON FUND was X. (2) The total number of TOKENS YOUR GROUP INVESTED IN THE COMMON FUND was X. (3) Your earnings previous to the group ranking multiplier were X. (4) Your group ranking was X. (5) Your earnings after the group ranking multiplier were X. (6) Based on your earnings, your ranking among the N participants was X. (7) Among the N participants the minimum earning of the round was X. (8) Among the N participants the maximum earning of the round was X.* The value N corresponded to the total of participants in the session.

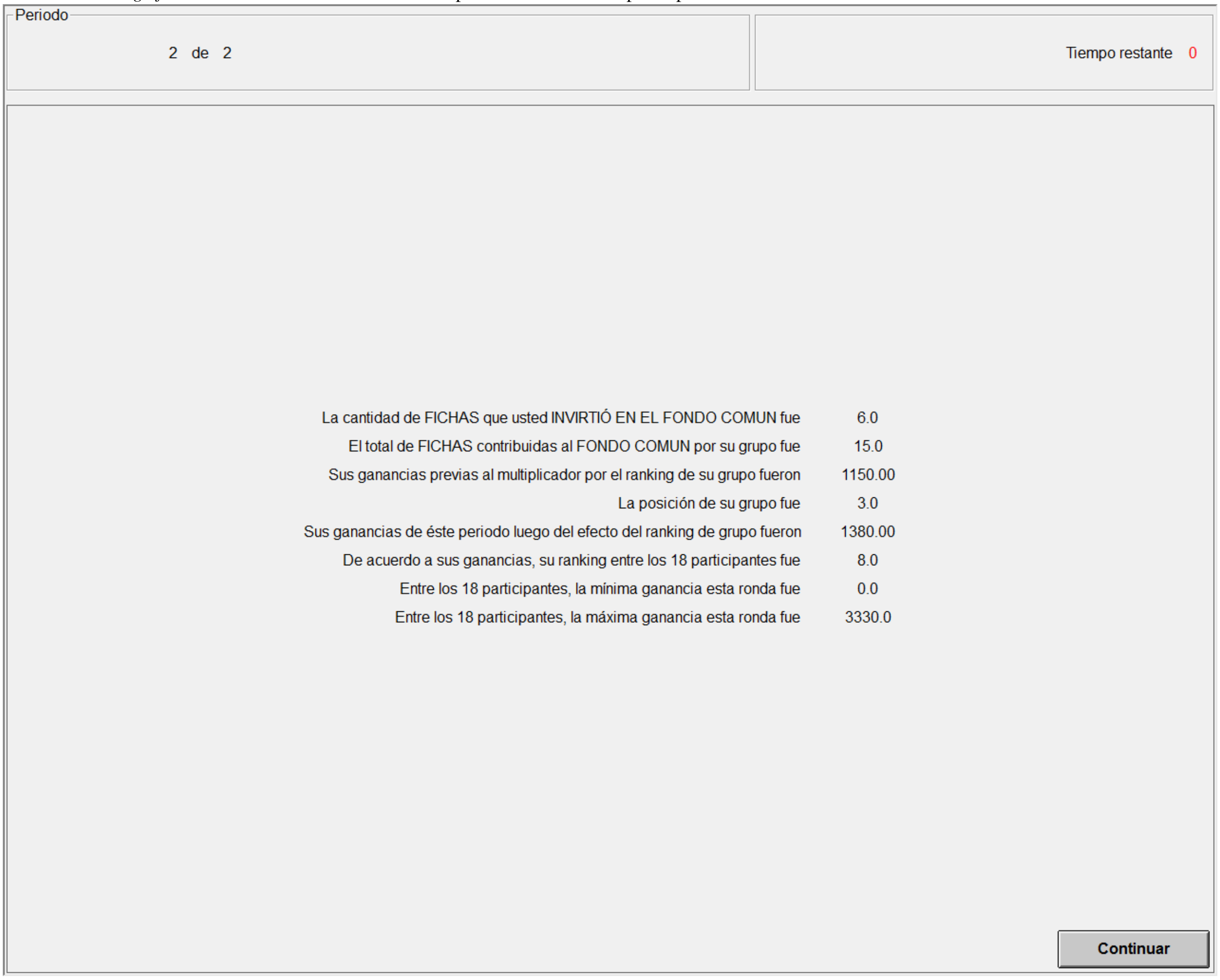

2 SUPPLEMENTARY TABLES

Table B.1 Alternative specification for the fixed effects regression: ΔContribution explained by individual and group rankings. Differences between treatments are statistically compared using the dichotomous variable *Competition*, which takes the value 0 in the *pseudocompetition* stage and the value 1 in the *competition* stage. Standard errors were computed clustering observations at the session level. For treatments with 18 and 24 participants the individual ranking was rescaled to match the treatment with 30 participants. The *Round* variable goes from 2 to 10 for each treatment.

Dependent variable: Δ Contribution	(1)	(2)
Competition	4.431***	7.681***
	(0.385)	(0.821)
Lagged Group Ranking	-0.626**	0.001
	(0.167)	(0.270)
Competition × Lagged Group Ranking	-5.754***	-8.594***
	(0.832)	(0.941)
Lagged Group Ranking Squared	0.018	-0.058
	(0.019)	(0.032)
Competition × Lagged Group Ranking Squared	0.454***	0.761***
	(0.090)	(0.105)
Lagged Individual Ranking	0.410***	0.404***
	(0.038)	(0.043)
Competition × Lagged Individual Ranking	0.892***	1.149***
	(0.149)	(0.137)
Lagged Individual Ranking Squared	-0.007***	-0.006***
	(0.001)	(0.001)
Competition × Lagged Individual Ranking Squared	-0.015***	-0.020***
	(0.004)	(0.003)
Round	-0.012	-0.010
	(0.024)	(0.024)
Competition × Round	-0.072**	-0.058
	(0.025)	(0.043)
Constant	-2.601***	-3.794***
	(0.360)	(0.571)
Group ranked at bottom excluded	No	Yes
Observations	2,592	2,202
R-squared	0.231	0.281
Number of subjects	144	144

Clustered standard errors reported in parentheses. *** $p < 0.01$, ** $p < 0.05$, * $p < 0.1$

Table B.2. Robustness check for the fixed effects regression reported in Table ??: subjects playing the Nash Equilibrium in the previous round (*i.e.* constrained to modify their contribution) excluded from the sample. Standard errors were computed clustering observations at the session level. For treatments with 18 and 24 participants the individual ranking was rescaled to match the treatment with 30 participants. The *Round* variable goes from 2 to 10 in all regressions.

Dependent variable:	Pseudocompetition		Competition	
Δ Contribution	(1)	(2)	(3)	(4)
Lagged Group Ranking	-0.847***	-0.397	-6.716***	-9.321***
	(0.168)	(0.253)	(0.711)	(0.589)
Lagged Group Ranking Squared	0.016	-0.039	0.462***	0.746***
	(0.015)	(0.025)	(0.058)	(0.051)
Lagged Individual Ranking	0.418***	0.412***	1.293***	1.609***
	(0.064)	(0.063)	(0.136)	(0.119)
Lagged Individual Ranking Squared	-0.006**	-0.006**	-0.020***	-0.026***
	(0.002)	(0.002)	(0.003)	(0.002)
Round	-0.033	-0.051	-0.074**	-0.070
	(0.026)	(0.029)	(0.025)	(0.040)
Constant	-1.889**	-2.529**	2.626***	4.667***
	(0.552)	(0.811)	(0.621)	(0.611)
Group ranked at bottom excluded	No	Yes	No	Yes
Observations	1,043	922	814	646
R-squared	0.329	0.327	0.116	0.178
Number of subjects	143	143	129	129

Standard errors reported in parentheses. *** $p < 0.01$, ** $p < 0.05$, * $p < 0.1$

Table B.3. Fixed effects regression for subsamples of 18, 24 and 30 subjects per session: Standard errors were computed clustering observations at the session level. For treatments with 18 and 24 participants the individual ranking was rescaled to match the treatment with 30 participants. The *Round* variable goes from 2 to 10 in all regressions.

Dependent variable:	Pseudocompetition			Competition		
Δ Contribution	N = 18	N = 24	N = 30	N = 18	N = 24	N = 30
	(1)	(2)	(3)	(4)	(5)	(6)
Lagged Group Ranking	-1.130*	-1.132**	-0.963	-7.639*	-9.078***	-4.647**
	(0.104)	(0.0662)	(0.259)	(0.994)	(0.142)	(0.164)
Lagged Group Ranking Squared	0.045	0.043	0.035**	0.640	0.626**	0.217
	(0.050)	(0.016)	(0.001)	(0.103)	(0.012)	(0.070)
Lagged Individual Ranking	0.319	0.425	0.480	1.378	1.873***	1.025**
	(0.176)	(0.089)	(0.101)	(0.264)	(0.029)	(0.061)
Lagged Individual Ranking Squared	-0.003	-0.006	-0.008	-0.025	-0.030***	-0.013**
	(0.006)	(0.002)	(0.003)	(0.006)	(0.000)	(0.001)
Round	-0.008	0.029	-0.050	-0.061	-0.072	-0.109
	(0.079)	(0.020)	(0.015)	(0.011)	(0.022)	(0.050)
Constant	-1.434	-2.245	-1.930	3.329*	2.422***	1.482
	(1.857)	(0.832)	(0.551)	(0.311)	(0.009)	(0.309)
Observations	324	432	540	324	432	540
R-squared	0.409	0.463	0.351	0.143	0.248	0.117
Number of subjects	36	48	60	36	48	60

Standard errors reported in parentheses. *** $p < 0.01$, ** $p < 0.05$, * $p < 0.1$

Table B.4. Variance ratio test for changes in contributions for given lagged group and individual rankings.

	**Group Ranking (1 worst - 6 best)**					
	1	2	3	4	5	6
Std. dev. *pseudocompetition*	2.66	2.46	3.19	3.22	2.49	2.72
Std. dev. *competition*	4.06	3.86	3.10	2.22	1.31	1.24
Difference	-1.40***	-1.40***	0.09	1.00***	1.18***	1.48***

	**Individual Ranking (1 worst - 30 best)**					
	[1-5]	[6-10]	[11-15]	[16-20]	[21-25]	[26-30]
Std. dev. *pseudocompetition*	3.33	2.50	2.33	2.31	2.19	2.13
Std. dev. *competition*	4.05	3.89	3.15	2.25	1.6	1.16
Difference	-0.72***	-1.39***	-0.82***	0.06	0.59***	0.97***

*** $p < 0.01$, ** $p < 0.05$, * $p < 0.1$

3 QUESTIONS FROM THE POST EXPERIMENTAL SURVEY

The following six questions were used to measure the participant's preferences for competition. For the first five questions it was used a Likert scale with four levels (*Completely disagree*, *Disagree*, *Agree* and *Completely agree*). For the last question the three response options are displayed below.

1. Grades must be based only on individual performance, not on groupwork
2. Groupwork is unadequate because it generates incentives for free-riders
3. Groupwork allows to improve teamwork abilities
4. The abilities most rewarded in the job market are the individual ones
5. I'd rather having more approved/unapproved courses to not be worried by the grade
6. In a groupwork, which of the following grading schemes do you prefer?
   - The grade is based on individual performance
   - The grade is based on individual performance (50%) and on the average performance of the group (50%)
   - The grade is based on the average performance of the group
